# Managing invasive species

**DOI:** 10.12688/f1000research.15414.1

**Published:** 2018-10-23

**Authors:** Patrick C Tobin

**Affiliations:** 1 School of Environmental and Forest Sciences, University of Washington, Seattle, WA, USA

**Keywords:** Biological invasions

## Abstract

Invasive species pose considerable harm to native ecosystems and biodiversity and frustrate and at times fascinate the invasive species management and scientific communities. Of the numerous non-native species established around the world, only a minority of them are invasive and noxious, whereas the majority are either benign or in fact beneficial. Agriculture in North America, for example, would look dramatically different if only native plants were grown as food crops and without the services of the European honey bee as a pollinator. Yet the minority of species that are invasive negatively alter ecosystems and reduce the services they provide, costing governments, industries, and private citizens billions of dollars annually. In this review, I briefly review the consequences of invasive species and the importance of remaining vigilant in the battle against them. I then focus on their management in an increasingly connected global community.

## Invasive species: what is the big deal?

Invasive species have tremendous negative influence in native ecosystems, cultivated ecosystems, and managed landscapes. It is this negative influence that defines an invasive species and separates them from non-native species that are not considered to be invasive or noxious. The majority of non-native species introduced to a new area are relatively benign, pose only negligible impacts, or are beneficial ^1– 3^; yet, the minority of introduced species that are invasive cause billions of dollars of damage annually ^4– 7^. Some non-native species have clear and unambiguous negative impacts, such as those that require costly management interventions (that is, non-native agricultural crop pests ^8^) or cause the functional extinction of native species (that is, brown tree snake in Guam ^9^), whereas others have documented positive benefits to native ecosystems and provide important ecosystem services ^3^. However, quantifying negative impacts or the potential to cause negative impacts in many non-native species remains a challenge ^10^. After all, the definition of any “pest” species—invasive or native—is linked to human expectations, which differ among individuals ^11^. Recent work highlights both conceptual and experimental approaches to better assign and predict the impacts of non-native species ^10, 12^.

The first attempts to quantify invasive species impacts were undoubtedly motivated by the economic damage caused by invasive weeds, insect pests, and plant pathogens in agricultural commodities ^13^. The threat to agriculture has not subsided in recent years, and many global agricultural systems are still vulnerable to invasive species, particularly in developing countries where the costs of the impacts can be high relative to a country’s gross domestic product ^7^. Many earlier scientific studies on invasive species impacts often considered direct and singular impacts, such as the loss of a specific native species in response to the introduction of a specific non-native species. The functional extinction of the American chestnut ( *Castenea dentata*) following the introduction of a non-native pathogenic fungal pathogen ( *Cryphonectria parasitica*) in the eastern United States is a prime example ^14^. A recent study considered extinction from a broader perspective and used data from the International Union for Conservation of Nature Red List of Threatened Species to quantify the frequency that non-native species were cited as a cause of extinction in species of plants, amphibians, reptiles, birds, and mammals ^15^. The results were alarming; the authors observed that non-native species were cited as the cause in 124 of 215 extinct species, second only in cause to exploitation (125 of 215 extinct species) ^15^.

More recent studies on the effects of invasive species have considered their cascading effects, both direct and indirect. For example, in a global meta-analysis, researchers examined the role that invasive species played in decreasing native species richness and reported that even a single invading species can cause a 16.6% decrease in species richness; losses in species richness were noted in both terrestrial and aquatic habitats ^16^. Using a spatial analysis, the authors also observed that declines in native species richness in Europe that were due to invasive species were spatially autocorrelated; in other words, a decline in species richness from a local-scale study was similarly observed across a larger spatial scale ^16^. The ramifications of invasive species can also be expressed through food webs with consequences to ecosystem services. For example, the introduction of a single invasive species, the spiny water flea ( *Bythotrephes longimanus*), in the Laurentian Great Lakes resulted in a trophic cascade by reducing densities of a grazer ( *Daphnia pulicaria*), ultimately leading to a decline in water quality at a cost of $140 million (USD) ^17^. Another recent study used a global meta-analysis of invasive species in aquatic habitats and also reported strong negative impacts on aquatic communities ^18^. In a meta-analysis of the impact of invasive plants, researchers compiled 3,624 observations from 198 studies and reported that invasive plants significantly reduced animal abundance and had a reducing effect in 56% of cases, a neutral effect in 44% of cases, and no positive effects ^19^. Moreover, even when a non-native species is not necessarily invasive, there are documented cascading impacts through the ecosystem. For example, in an urban-based study, scientists reported that non-native plants reduced the abundance and diversity of the native herbivore caterpillar community ^20^, which had a cascading effect of reducing the abundance and diversity of birds, which consume caterpillars ^21^. The prominence of non-native plants in urban forest ecosystems, even when non-invasive, could contribute to a lack of biodiversity in these environments ^22^. Although some have argued that the problem of invasive species is often overblown given examples of native species that pose perhaps even more ecological damage than non-native species, non-native invasive species remain a great threat to ecosystem function and biodiversity ( 23 and references within).

## Invasive species: have we not studied this topic enough already?

**Figure 1.**
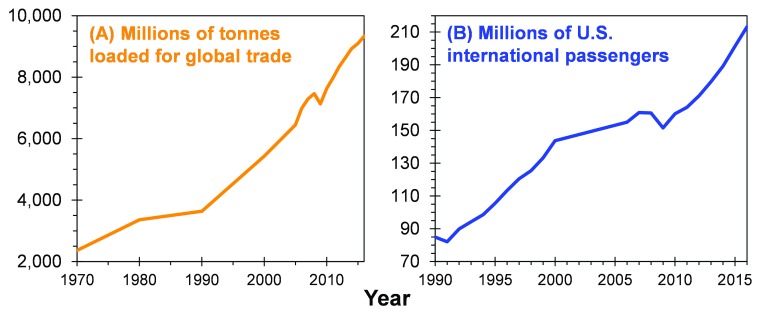
Trends in global trade and travel. ( **A**) Millions of tonnes loaded for global trade, 1970–2016 ^29^. ( **B**) Millions of international passengers departing from US airports, 1990–2016 ^30^.

Attention to the management of non-native, invasive species has a long history that predates academic work on the subject. In the United States, regulatory officials recognized the threat of non-native species to agricultural interests, leading to early efforts in classic biological control in the late 19th century ^24^ and eventually to the passage of the Plant Quarantine Act of 1912 (US Public Law 62-275). This Act empowered the Secretary of Agriculture to regulate the importation of nursery stock that could carry “injurious plant diseases and insect pests” that could be harmful to agriculture. Elton’s seminal book on biological invasions ^25^ paved the way for scientific study on the biology and ecology of invasive species, but it was not until the 1990s that citations of papers on invasion ecology began to increase exponentially ^26^. A current search in Google Scholar under the term “biological invasion” yields 7,160 results between 2015 and 2018 alone. Moreover, at some point, one might assume, given the extent of international trade and travel over the past several years ( Figure 1) as well as the long history of colonization around the world ^27^, that every non-native species capable of establishing outside of their native range has done so by now. Indeed, a cynical perspective might to be assume that there is little left to learn in the field of biological invasions at this point.

However, this is not yet the case. Invasions by terrestrial non-native species are often a consequence of hitchhiking on freight, shipping containers, or the body or interior of the ship ^28^ or being carried in airline baggage ^31^ or on imported plants ^32^. Moreover, ballast water is a well-known vehicle of aquatic species movement, and at any given time, about 10,000 aquatic species are thought to be transported in ballast water tanks alone ^33^. Consequently, under an international maritime treaty on ballast water management, which was adopted in 2004 and implemented in 2017, cargo ships of signatory countries are required to have a ballast water management plan to limit the introduction of non-native aquatic organisms ^34^. Regardless, given the steady increase in global trade and travel ( Figure 1), there is no reason to assume that species introductions will decline. Also, traditional global trade pathways (that is, imported freight on cargo ships) do not include all potential invasion pathways. For example, the movement of invasive species through internet-based commerce, such as eBay ^®^^35^, is historically poorly regulated. There has been attention to the importance of the pet trade industry as a pathway through which non-native species, such as aquaria and other exotic pets, are imported ^36, 37^, which requires better enforcement, including financial penalties applied to the “polluter” ^38^. The introduction and subsequent establishment of Burmese pythons ( *Python molurus*) in Florida through the pet trade are examples of the ecological problems posed by this pathway ^39^. Moreover, the presence of currently legal purchases of potentially invasive species, such as biological organisms for use in school science curricula, remains a largely unregulated invasion pathway ^40^.

In addition, recent data suggest that the accumulation of non-native species has not reached a plateau ^41^. The authors compiled a global database of 16,926 established non-native species across taxa from 1500 to 2014 and noted that most arrived to a new area during the last 200 years but that over one third of species arrived to a new area from the 1970s to 2014 ^41^. A recent study from California showed that each year, about nine non-native species arrive and successfully establish in the State, up from about six per year from 1970 to 1989 ^42^. Although many plant introductions have been intentional (even in plants that end up as invasive, for example, Kudzu, Japanese knotweed), van Kleunen *et al*. ^43^ showed that while 13,168 plant species have been successfully introduced outside of their respective native ranges, this number represents only 3.9% of the extant vascular flora in the world. Thus, the number of plant species that can still be introduced into novel areas remains quite large. Lastly, a global analysis of the threats from invasive species suggested that one sixth of the land surface of the Earth is very susceptible to invasion, particularly in developing countries where the infrastructure to respond could be limited or lacking ^44^. This evidence suggests that the study of invasive species is far from being complete or passé.

## Invasive species: what can we do about it?

Fortunately, most non-native species are thought to fail to establish after arriving to a new location; this is for many reasons, including a failure to survive the journey, climate mismatch, insufficient food resources at the port of entry, and insufficient founder population size ^26^. It is nearly impossible to estimate how many arriving species fail because they often fail without human knowledge of their failure. However, prior studies have conservatively suggested that only a minority of arriving species successfully establish ^45– 47^ and even fewer of those are ultimately considered to be invasive ^48^. However, with the continual arrival of non-native species owing to global trade and travel, society will have to continue to deal with a known unknown of biological invasions; that is, we know non-native species will continue to be introduced into new areas, but we do not know which ones will be invasive and where they will be invasive. Thus, the question of what to do about it remains an important topic of discussion. Compounding the problem is that even in developed nations, resources for preventing the arrival stage of non-native species are limited; for example, only about 2% and about 10% of inbound cargo are inspected for non-native species in the United States ^49^ and New Zealand ^50^, respectively.

However, there have been recent advances in efforts to manage invasive species. Paramount to the development and implementation of effective management strategies against invasive species is the consideration of the stage of the invasion process being addressed ( Table 1). One effective strategy is to prevent a species from arriving in the first place, and recent work involving risk analyses has helped to refine estimates of likely invasion pathways and the time at which the pathway is most likely to result in successful establishment. For example, Gray ^51^ developed a decision support model that considers the phenology of an insect pest in its native area, the probability that the most transportable life stage (for example, the one most likely to survive the trip) will be accidently brought on board, and the shipping route and schedule to optimize the allocation of inspection resources given that such resources are finite. Researchers have also highlighted the complexities in managing invasion pathways and the need for government resources dedicated to developing risk assessments for species before and after they arrive to a new area and the need for industry and consumer cooperation and education ^52^. Advances in risk analyses also include linking biological information, as well as the use of new technologies for detection and surveillance, such as environmental DNA ^53^, with bioeconomic models to address the costs of different management strategies ^54, 55^. Lastly, species distribution models can be used to predict susceptible areas for an invading species on the basis of biological aspects of the organism and climate suitability ^56^, although this approach has been criticized for lacking validation ^57^.

**Table 1.** Stages of the biological invasions ^26^ and the potential management strategies for each stage ^60^.

Stage of invasion	Management strategies
Arrival	Risk analysis International standards Inspection
Establishment	Detection Eradication
Spread	Quarantine Barrier zone
Impact	Suppression Adaptation

In the event of a failure to exclude a non-native species from arriving, early detection–rapid response programs become a critical element, especially if eradication is the management goal. Eradication becomes a less biologically and economically feasible option as the species occupies more area and if detection methods are unreliable ^58, 59^. The role of citizen scientists and their engagement in the management of invasive species should not be overlooked given that management resources will always be a limiting constraint. Criticisms of citizen scientists often include the lack of credibility by non-scientists and their collective inability to distinguish, especially in the absence of taxonomic dichotomous keys or molecular methods, between native and non-native species. Indeed, even learned scientists can be challenged to identify an individual organism to the level of species, especially when it is a newly established species. In a recent study, scientists demonstrated how data from citizen scientists regarding invasive plants can be useful in filling knowledge gaps ^61^, especially with regard to their distribution given the extent to which citizen scientists can sample in areas otherwise not sampled ^62^. Undoubtedly, a level of training is required, and the development of technologies such as phone-based apps to report and upload photos and georeferenced information of suspected non-native species, which in turn can be verified by experts, provides both a medium for engaging citizen scientists and a process of quality control ^63, 64^.

Managing invasive species in urban landscapes has been at the forefront for the past few decades ^65^ yet remains a challenge given the interconnected role between government agencies charged with their management and private citizens who live in these areas. Not only is the world becoming increasingly connected through global trade and travel, but human populations are becoming more urban. In the United States, more than 80% of the population reside in an area designated as urban ^66^, which brings unique challenges to invasive species management. Moreover, owing to trade and travel pathways, urban areas with international airports and shipping ports are often the first place of arrival for non-native species. Some of these challenges include the costs, particularly with regard to the increased liability to municipalities when urban trees are killed by invasive species. For example, a recent study showed that in cities the majority of all management costs due to invasive insect wood bores, such as the emerald ash borer ( *Agrilus planipennis*), are due to the costs of hazard tree removal ^67^. The same trends in costs have been shown to be the case for invasive plant defoliators and invasive plant pathogens in urban environments ^68, 69^.

However, costs represent only some of the challenges associated with invasive species, and increasingly researchers have noted the social dimensions of invasive species management ^70^. Using Cape Town, South Africa as an example municipality, a recent paper outlined a framework of invasive species management that includes greater attention to stakeholders, such as the public, in the decision-making process ^71^. Public stakeholders should not be overlooked; for example, Estévez *et al*. ^72^ examined more than 15,000 publications on biological invasions in the peer-reviewed literature and noted that while only 124 publications considered the social dimensions of the biological invasion process (a problem in itself), about 23% included reports of contentious situations. The authors observed that the cause of the conflicts was due mostly to the variability in the value systems among different groups ^72^. Similarly, Woodford *et al*. ^73^ noted that implementation of successful management strategies against invasive species was affected by the disconnect between the perception of the problem, which can vary depending on the viewpoints of different stakeholder groups, and the reality of the problem.

## Managing invasive species: where do we go from here?

Management decisions against invasive species, regardless of the stage of the invasion process or the strategy, are not trivial. In the United States, for example, the decision process often includes scientific advisory committees, public outreach, and a public commentary period ^74, 75^. However, recent controversies over proposed management strategies against the light brown apple moth ( *Epiphyas postvittana*) in California ^76, 77^ and the Asian carp in Illinois and Michigan ^78, 79^ demonstrate that there is still an opportunity to improve the management process. Undoubtedly, the largest elephant in the room is us and our general lack of compliance or lack of awareness of the problem of invasive species or both.

For example, recall the story of the actress Hilary Swank, who, not long after winning her second Academy Award, for her lead role in *Million Dollar Baby*, brought an apple and orange on a flight to New Zealand, failed to declare them upon entry, and was subjected to a fine for violating the biosecurity regulations of New Zealand ^80^. Although no one could fault Ms. Swank for packing a snack for a long international flight, the probability of accidently introducing an invasive pest or pathogen likely never occurred to her, nor would it likely occur to the vast majority of airline passengers. Indeed, a prior study provided evidence of industry compliance with regulations designed to limit the movement of invasive species, whereas the public was seemingly non-compliant with (or likely simply unaware of) the same regulations ^81^. Yet the majority of the costs of invasive species are shouldered by the general public and local governments ^4^. There are also opportunities to improve the management and compliance of certain pathways, most notably the horticultural plant pathway ^82^. Most invasive plants were originally introduced as ornamental plants ^83^ and are also recognized as a vector on which invasive insects and pathogens can be introduced ^32^. As humans continue to crave plants for their gardens and dwellings, this pathway will continue to be an important avenue of invasive species introduction. Education efforts that target the horticultural industry, especially with regard to the sale of plants that are known to be invasive, are still needed ^84^, but the lack of knowledge of the invasive species problem by the general public remains a formidable obstacle.
